# Medication Assessment in an Older Population during Acute Care Hospitalization and Its Effect on the Anticholinergic Burden: A Prospective Cohort Study

**DOI:** 10.3390/ijerph20075322

**Published:** 2023-03-30

**Authors:** Mariona Espaulella-Ferrer, Nuria Molist-Brunet, Joan Espaulella-Panicot, Daniel Sevilla-Sánchez, Emma Puigoriol-Juvanteny, Marta Otero-Viñas

**Affiliations:** 1 Servei Territorial de Geriatria i Cures Pal·Liatives d’Osona i el Ripollés, Hospital Universitari de la Santa Creu de Vic, Hospital Universitari de Vic, 08500 Vic, Spain; 2 Tissue Repair and Regeneration Laboratory (TR2Lab), Institut de Recerca i Innovació en Ciències de la Vida i de la Salut a la Catalunya Central (IRIS-CC), 08500 Vic, Spain; 3 Central Catalonia Chronicity Research Group (C3RG), Institut de Recerca i Innovació en Ciències de la Vida i de la Salut a la Catalunya Central (IRIS-CC), 08500 Vic, Spain; 4 Pharmacy Department, Parc Sanitari Pere Virgili, 08023 Barcelona, Spain; 5 Epidemiology Department, Hospital Universitari de Vic, 08500 Vic, Spain; 6 Multidisciplinary Inflamations Research Group (MIRG), Institut de Recerca i Innovació en Ciències de la Vida i de la Salut a la Catalunya Central (IRIS-CC), 08500 Vic, Spain; 7 Faculty of Science, Technology and Engineering, University of Vic-Central University of Catalonia (UVic-UCC), 08500 Vic, Spain

**Keywords:** frailty, polypharmacy, anticholinergic burden, drug burden index, medication review

## Abstract

(1) Background: Anticholinergic and sedative drugs (ASDs) contribute to negative health outcomes, especially in the frail population. In this study, we aimed to assess whether frailty increases with anticholinergic burden and to evaluate the effects of medication reviews (MRs) on ASD regimens among patients attending an acute care for the elderly (ACE) unit. (2) Methods: A cohort study was conducted between June 2019 and October 2020 with 150 consecutive patients admitted to our ACE unit. Demographic, clinical, and pharmacological data were assessed. Frailty score was determined using the Frail-VIG index (FI-VIG), and ASD burden was quantified using the drug burden index (DBI). In addition, the MR was performed using the patient-centered prescription (PCP) model. We used a paired T-test to compare the DBI pre- and post-MR and univariate and multivariate regression to identify the factors associated with frailty. (3) Results: Overall, 85.6% (*n* = 128) of participants showed some degree of frailty (FI-VIG > 0.20) and 84% (*n* = 126) of patients received treatment with ASDs upon admission (pre-MR). As the degree of frailty increased, so did the DBI (*p* < 0.001). After the implementation of the MR through the application of the PCP model, a reduction in the DBI was noted (1.06 ± 0.8 versus 0.95 ± 0.7) (*p* < 0.001). After adjusting for covariates, the association between frailty and the DBI was apparent (OR: 11.42, 95% (CI: 2.77–47.15)). (4) Conclusions: A higher DBI was positively associated with frailty. The DBI decreased significantly in frail patients after a personalized MR. Thus, MRs focusing on ASDs are crucial for frail older patients.

## 1. Introduction

The world population is aging, and the numbers of very old people are increasing even faster. Aging is associated with the onset of frailty, which is a biological syndrome involving decreased reserves and resistance to stressors resulting from cumulative declines across multiple physiologic systems and causing vulnerability to adverse outcomes [1,2]. In addition, aging is often related to increases in comorbidities, which produce changes in overall health status. Multimorbidity (the presence of two or more chronic conditions) is often associated with polypharmacy and exposure to anticholinergic and sedative drugs (ASDs) [3,4,5].

ASDs have been linked to the presence of frailty [6,7,8]. This kind of medication is frequently administered to older patients, and previous studies estimated the prevalence of the use of these anticholinergic drugs in older patients at between 37% and 63% [9]. Their negative effects are known to affect multiple organs in the body and, in the case of older people, are more likely to lead to anorexia, constipation, urinary retention, falls, cardiovascular effects, poor vision, and mortality [10,11]. Moreover, anticholinergic drugs have systemic effects that could contribute to functional and cognitive impairment [11,12,13].

Since ASDs can contribute to negative health outcomes, it is essential to pay special attention to this group of medications, the cumulative effects of which are known as the anticholinergic burden. There are many risk scales that can be used to assess anticholinergic burden, but only the drug burden index (DBI) considers the daily dose administered [14]. Previous studies have demonstrated a higher rate of admissions among patients subjected to anticholinergic burden [15].

Very old patients need more frequent clinical care and, during hospitalization, tend to present more complications and greater functional decline [16]. Thus, they benefit from admission to special wards, such as acute care for the elderly units. One of the most frequent interventions upon admission is the medication review (MR), which consists of a structured examination of patients’ prescriptions focused on optimizing medication use and improving health outcomes [17,18]. Prescribing for these complex patients appropriately is challenging and cannot be resolved by simply applying clinical practice guidelines [19,20]. This is a high-value practice, as scientific evidence suggests that medication use in elderly patients is often inappropriate [21,22], increasing the risk of this group of people suffering adverse drug events [3,4,21,23,24,25,26]. Previous studies estimated that between 5 and 20% of admissions among people aged ≥70 years are drug-related [27]. Thus, admission is an appropriate moment to perform a complete review of the patient’s therapeutic plan, and doing so from a patient-centered viewpoint is essential [28,29].

The MR should consider pharmacological and clinical aspects, as well as the patient’s care goals. The MR can be carried out with a quantitative vision, which considers diminishing the number of daily chronic drugs, and a qualitative vision, where the emphasis is placed on the drugs that may generate more negative effects, such as ASDs. To achieve the appropriate medication regimen, an MR should be systematic and periodic [30].

Molist-Brunet et al. recently reported a patient-centered prescription (PCP) model for the improvement of MRs. This PCP model is a four-stage process performed by a multidisciplinary team composed of a geriatrician, a clinical pharmacist, and a nurse. The model consists of a review of the medication focused on the pharmacological plan, comorbidities, and the global health status of the individual [31,32,33,34,35].

In this study, we hypothesized that anticholinergic and sedative burden would be associated and increase with the degree of frailty in patients admitted to an ACE unit. In addition, an MR protocol considering the anticholinergic medication regimen may benefit this group of patients. Thus, our main objectives included: ascertaining the most widely prescribed medications creating anticholinergic and sedative burden among patients admitted to an acute care for the elderly (ACE) unit, evaluating the association between anticholinergic and sedative burden and frailty, and verifying whether appropriate prescription proposals are maintained after three months of follow-up.

## 2. Materials and Methods

### 2.1. Study Design and Subjects

This open, prospective, cohort study was carried out in a medical–surgical ACE unit at a secondary care hospital, and the participants were part of a cohort of patients who live in the community (Community Older Patients (COP) cohort) [32].

**Figure 1:**
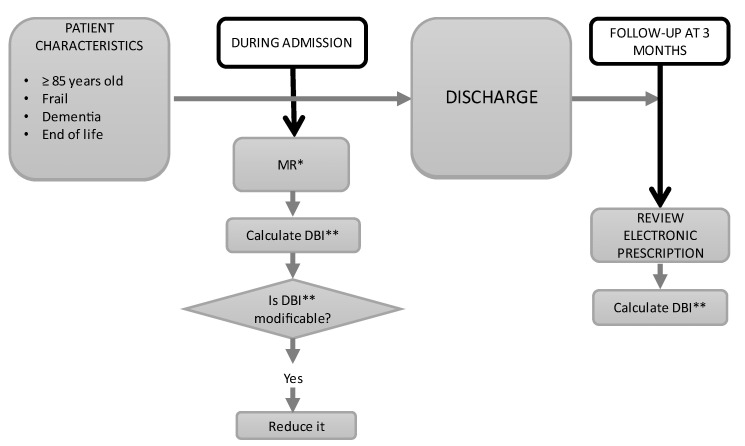
Overview of the interventions and assessment. * MR: medication review; ** DBI: drug burden index.

Figure 1 shows an overview of the interventions and assessment included in this study. Inclusion criteria for admission to the ACE unit were: (a) age 85 or older, (b) presence of cognitive impairment, or (c) presence of advanced chronic disease. Exclusion criteria were: patients who died within the first 48 h of admission or were discharged before 48 h had elapsed. We included consecutive patients admitted to our ACE unit who received an MR applying a PCP model considering the assessment of the anticholinergic and sedative burden between June 2019 and October 2020.

Upon admission, we analyzed chronic medications, and the DBI was calculated (DBI pre-MR) for each patient. Afterward, an individualized MR was performed according to the administered medication. During hospitalization, the MR proposals were agreed upon with the patient/caregiver, implemented in the treatment plan, and kept after discharge. These proposals were communicated to the patient’s general practitioner in the discharge summary. Three months after discharge, we verified the persistence of the changes proposed at admission in the electronic prescription, and the DBI post-MR was determined.

### 2.2. Sample Size

A sample of 150 individuals was sufficient to estimate—with 95% confidence and ±8% precision—the percentage of anticholinergic load that was predicted to remain as approximately 66%, estimating 10% loss to follow-up.

### 2.3. Pharmacological Assessment and Intervention

**Figure 2:**
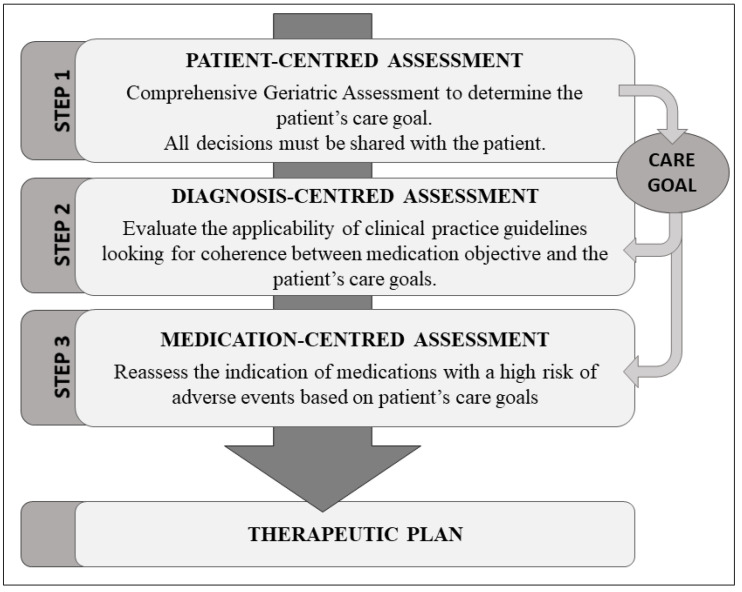
Patient-centered prescription (PCP) model.

The pharmacological assessment and intervention were performed using the PCP model. The PCP model is a four-step model based on a comprehensive geriatric assessment (CGA) that integrates the calculation of a frailty index (Frail-VIG index) [36]. Daily, a multidisciplinary team of geriatricians and a clinical pharmacist reviewed the medication plans of each patient and applied the PCP model [32,34,35], which focuses therapeutic decisions on the global assessment of the patients and their individual therapeutic goals (prolonging survival, improving or maintaining function, or symptomatic control) [37]. All decisions were made in agreement with the patient or the patient’s main caregiver if the patient lacked decision-making capacity (Figure 2).

### 2.4. Outcome Measures

Patients’ anticholinergic burden was determined using the DBI [14]. The DBI is a pharmacological risk assessment tool that calculates the anticholinergic and sedative effects of all chronic medications. Interestingly, this instrument takes into consideration the dose of the drug. DBI scores were categorized into three groups according to the total score: <1 point (low anticholinergic burden), 1–2 points (moderate anticholinergic burden), and >2 points (high anticholinergic burden).

The data collected included the following parameters:

(a) Sociodemographic data: age, gender, and place of residence;
(b) Functional data (patient’s global health status): daily living activities quantified with the Barthel index [38]; various instrumental activities of daily living, such as handling finances, using a phone, and handling medications; and geriatric syndromes, such as falls, delirium, insomnia, dysphagia, constipation, urinary incontinence, and malnutrition [39,40];
(c) Clinical data: comorbidities assessed using expanded diagnostic clusters with the Johns Hopkins University ACG System and Charlson Index [41] and cognitive assessment according to the Global Deterioration Scale—Functional Assessment Staging (GDS-FAST) for Alzheimer’s disease or the Clinical Dementia Rating scale in the case of other types of dementia [42];
(d) Frailty: frailty was evaluated using the Frail-VIG index [36,43], which is based on a CGA and includes 25 items that assess functionality, cognition, social status, geriatric syndromes, and comorbidities. The Frail-VIG index enables the classification of patients into four groups according to their Frail-VIG scores: 0–0.19 (non-frail), 0.20–0.35 (mildly frail), 0.36–0.49 (moderately frail), and >0.50 (severely frail). In some analyses, the frailty variable was valued as a binary variable: non-frail (Frail-VIG score of 0–0.19) vs. frail (Frail-VIG score ≥ 0.20);
(e) Complexity identification: chronic complex patients (CCPs) were defined as individuals in situations that included difficulties in their management and care, who needed to adopt specific individual plans owing to concurrent diseases, or who experienced difficulties in their utilization of healthcare services or in relation to their context according to Catalan Health Department criteria [44]. Identification of end-of-life (EOL) patients was undertaken using the NECPAL CCOMS-ICO^©^ tool criteria [45]. These were patients who were considered to be in the final months or year of their life. The criteria used to identify them as EOL patients included: (a) identification as such by their primary care physician, (b) advanced disease criteria [45], or (c) Frail-VIG index > 0.50;
(f) Therapeutic goals: in accordance with their baseline situation, patients were classified into groups with different goals: survival for patients with a robust baseline situation; functional for patients with an intermediate situation; and symptomatic for vulnerable patients (including end-of-life patients);
(g) Pharmacological data: these data included the prevalence of polypharmacy (≥5 drugs) and severe polypharmacy (≥10 drugs) [20]. An MR was carried out to detect inappropriate prescriptions (IPs) among the most prevalent chronic conditions using current evidence and by applying the latest guidelines and recommendations issued by scientific societies [31,33,34]. Therapeutic complexity was qualified according to the Medication Regimen Complexity Index (MRCI) [46].

### 2.5. Ethical Considerations

All clinical procedures involved in this study were in accordance with the institutional guidelines and were approved by the hospital ethics committee (PR237). Researchers undertook to protect patient privacy and the procedures in this study were in accordance with the Declaration of Helsinki.

### 2.6. Statistical Analysis

Results for categorical variables were expressed as absolute and relative frequencies and results for continuous variables as means and standard deviations (SDs). The relationships between the different variables studied and the DBI were analyzed using the chi-squared test when the variables remained categorical (with expected frequencies below 5 in 2 × 2 tables or with Fisher’s exact test or Yates’s correction for other tables) or using Student’s *t*-test or ANOVA when one variable was quantitative and the other qualitative. Comparisons between the DBI pre- and post-MR were performed using Student’s *t*-test for paired data with parametric continuous variables. Univariate and multivariate logistic regression analyses were performed to identify factors associated with frailty. For the multivariate model, variables that had a *p*-value < 0.10 in the bivariate analyses were used with a forward stepwise regression model.

The level of statistical significance was set at *p* < 0.05. Statistical analyses were performed with the IBM SPSS Statistics v28.0 package (Armonk, NY, USA).

## 3. Results

### 3.1. Baseline Characteristics of the Study Population

A total of 150 patients, 25.3% (*n* = 38) from a nursing home, were included in the study. During the 3 month follow-up, 39 patients died and 2 patients were lost to follow-up. Table 1 shows the patients’ baseline characteristics. A total of 62% (*n* = 93) of patients were female. Patients’ mean age was 89 ± 4.5 years for men and 89.5 ± 4.3 years for women. High prevalences of various degrees of cognitive impairment (51.3%, *n* = 77) and functional dependence (82.7%, *n* = 124) were observed. Most of the patients had geriatric syndromes, the most prevalent being insomnia/anxiety, falls, and urinary incontinence. A total of 24.7% (*n* = 37) of patients presented with delirium in the six months prior to admission, and this condition rose to 45.3% (*n* = 68) during hospitalization.

A total of 66.7% (*n* = 100) of patients had five or more comorbidities (Table 1). The mean age-adjusted Charlson Comorbidity Index score was 2.83 ± 2.19 points. Frailty evaluation showed that 14.6% of patients (*n* = 22) were not frail (FI-VIG: <0.20), 32% (*n* = 48) were mildly frail (FI-VIG: 0.20–0.35), 36.7% (*n* = 55) were moderately frail (FI-VIG: 0.36–0.50), and 16.7% (*n* = 25) were severely frail (FI-VIG: >0.50) (Table 1).

### 3.2. Medication Review

After a CGA, the patients were classified into the following groups according to the therapeutic goals: survival (19.3%), maintenance of functionality (52.7%), and symptomatic control (28.0%). Table 1 lists the baseline pharmacological data. On average, prior to admission, patients were taking 8.7 ± 3.8 medications and only 16.7% (*n* = 25) of patients were not receiving polypharmacy. A total of 89.3% (*n* = 134) of patients had at least one inappropriate prescription (IP), and the IP mean was 3.37 ± 2.56 drugs. After three months of follow-up, 39 patients had died and 2 patients were lost to follow-up. The pre–post analysis of the results of the MR was performed with 109 patients. We put forward several medication review proposals by applying the PCP model; after three months follow-up, 79.4% of proposals were maintained (79.7% of medications had a DBI score = 0 vs. 78.0% of medications had a DBI score > 0, with no statistical differences (*p* = 0.786)). Daily consumption of medications decreased from 8.7 ± 3.8 to 7.8 ± 3.4 (*p* < 0.001). In addition, the medication complexity regimen decreased from 32.7 ± 16.1 upon admission to 29.1 ± 14.6 post-MR (*p* < 0.001). The most commonly prescribed medications were omeprazole, paracetamol, and furosemide at 61.3% (*n* = 92), 57.3% (*n* = 86), and 41.3% (*n* = 62), respectively. The most prescribed medications with anticholinergic or sedative effects were lorazepam, citalopram, and quetiapine at 44% (*n* = 66), 17.3% (*n* = 26), and 16.6% (*n* = 25), respectively.

### 3.3. Anticholinergic and Sedative Burden

Using the DBI score, we observed that 84% (*n* = 126) of patients received treatment with anticholinergic or sedative effects. After a 3 month follow-up, the mean DBI score decreased from 1.06 ± 0.7 to 0.95 ± 0.7 (*p* < 0.001).

Our results showed that, upon admission, the DBI score increased as the degree of frailty increased (*p* < 0.001), and this distribution was also observed at 3 months post-MR (Table 2). When we compared the DBI score pre- and post-MR, we observed a reduction in the DBI score in frail patients vs. non-frail patients (frail patients pre-MR: 1.16 ± 0.71 vs. post-MR: 1.02 ± 0.68, *p* < 0.001; non-frail patients pre-MR: 0.46 ± 0.52 vs. post-MR: 0.48 ± 0.48, *p* = 0.64).

In the multivariate age-adjusted study, our results showed that the factors associated with frailty were gender, the Barthel index, and the DBI (OR: 7.37 (95% CI: 1.5–36.2), OR: 0.90 (95% CI: 0.85–0.95), and OR: 11.42 (95% CI: 2.8–47.2), respectively) (Table 3).

As Table 4 shows, patients with no dementia had a lower DBI score in comparison to the dementia group (*p* < 0.001) and, interestingly, the group of patients with advanced dementia showed a high DBI score that increased up to 31% (*n* = 9).

## 4. Discussion

This prospective observational study showed a high prevalence of anticholinergic and sedative burden in a very old population. Furthermore, it demonstrated that frailer patients are at risk of a higher anticholinergic and sedative burden. Thus, our results demonstrate the risk of side effects from these medications [47,48,49]. In addition, we demonstrated that an overall MR using the PCP model leads to a decrease in the DBI in patients admitted to the ACE unit, and these changes persisted after three months.

In accordance with the population usually admitted to the ACE unit, our study included frail patients of very advanced age presenting multimorbidity, moderate dependence, and some degree of cognitive impairment [43]. Most of these subjects received polypharmacy and almost half of them had a moderate or high DBI score. In addition, this group of patients presented moderate therapeutic complexity and a mean of 3.37 IPs per patient. Similar polypharmacy results were reported by Antonio San Jose et al. in a study carried out in 2015 among oldest old Spanish patients admitted to hospital, although our data presented a higher number of IPs [50].

Furthermore, our results showed an association between frailty and anticholinergic and sedative burden. According to the literature, the frailer patients are, the more inappropriate anticholinergic medications are due to the higher risk of complications [12]. Both frailty and a high anticholinergic burden can lead to poor health outcomes, so their identification should be a trigger for an MR. Post-MR, our data confirmed a decrease in the DBI score among frail patients that was not seen in non-frail patients. This probably occurred because of the bidirectional relationship that exists between frailty and anticholinergic and sedative burden [51].

The very old population commonly suffers from disabilities induced by hospitalization [52], so it is strongly recommended that specific practices are identified that can contribute to achieving health benefits during hospitalization. One of these value-based practices is the MR [53,54]. The PCP model is an accurate MR protocol that considers the therapeutic objective, medication history, patient information, and clinical information, allowing the therapeutic plan to be individualized [32]. Our results from the application of the PCP model during hospitalization demonstrated that this intervention could reduce the total pharmacological burden, DBI scores, and therapeutic complexity, in concordance with other studies using the PCP model with older populations. In a recent study concerning community-dwelling populations, Molist-Brunet et al. reported that more than 90% of patients received at least one IP and, by applying the PCP model, they were able to optimize therapeutic plans by reducing polypharmacy, complexity, and anticholinergic and sedative burden [34].

After using the PCP model, 79.4% of our suggested changes to the therapeutic plans were implemented and persisted at 3 months of patient follow-up. The success of the de-prescribing process was due to several reasons. Firstly, the PCP model leads to the individualization of therapeutic plans by setting care goals and making shared decisions with patients and their caregivers, which generates a good relationship and good communication. Secondly, the recommendations are detailed in the discharge summary, which allows fluid communication with other levels of care. Both strategies have been shown in previous studies to be facilitators for de-prescription [55].

One of the strengths of this study was its improvement of prescription during hospitalization in an ACE unit, where patients are admitted for acute illnesses and have short stays.

In this study, patient mortality was higher than expected at the 3 month follow-up. This result may be explained by the fact that the data were collected during the COVID-19 pandemic, a period in which an increase in deaths was observed due to this disease [56].

Our study had some limitations. We only followed up patients for 3 months; this short time did not allow us to ascertain whether patients who received the MR intervention showed improved health outcomes, such as a decrease in the number of hospitalizations related to pharmacological events, a reduction in falls, or improvements in their cognition. Generalizing the application of the PCP model requires joint work between pharmacists and geriatricians, and this relationship is not implemented in all hospitals. We know that anticholinergic and sedative prescriptions can be harmful to patients but, to treat some conditions, such as an overactive bladder, we do not have a better alternative to replace them. In addition, we performed the MR according to the administered medication when patients were recruited and, at the 3 month follow-up, we verified the persistence of the proposed changes in the electronic prescription without analyzing the medication compliance in both situations. Finally, to facilitate daily practice, the use of electronic systems that automatically calculate the DBI and the frailty index would be very useful [55].

## 5. Conclusions

We strongly recommend systematically performing MRs with all patients admitted to ACE units. All patients are candidates for MRs based on their therapeutic goals. However, further studies are needed to confirm whether improving prescriptions correlates with better health outcomes, such as reducing frailty or diminishing geriatric syndromes. In addition, considering the results of our study, a reduction in anticholinergic and sedative burden might portend a decrease in frailty, as both situations are related to poor health outcomes. Further studies are needed to confirm this hypothesis.

## Figures and Tables

**Table 1:** Patients’ baseline data.

Baseline Data		Total N = 150
**Demographic Data**		
Age, mean (SD)		89.3 (4.4)
Gender, N (%)	Men	57 (38%)
	Women	93 (62%)
Origin, N (%)	Home	112 (74.7%)
	Nursing home	38 (25.3%)
**Clinical, Functional, and Cognitive Data**		
Medication self-management *		42 (37.5%)
Barthel index (BI), mean (SD)		59.8 (27.9)
Barthel index	Independence: BI ≥ 95	26 (17.3%)
	Mild dependence: BI 90–65	46 (30.7%)
	Moderate dependence: BI 60–25	59 (39.3%)
	Severe dependence: BI ≤ 20	19 (12.7%)
Cognitive status	No dementia	73 (48.7%)
	Mild dementia	22 (14.7%)
	Moderate dementia (from GDS 5 to GDS 6B)	26 (17.3%)
	Advanced dementia (from GDS 6C)	29 (19.3%)
Geriatric syndromes (GSs), mean (SD)		3.13 (1.6)
Type of GS	Falls	58 (38.7%)
	Dysphagia	28 (17.3%)
	Constipation	55 (36.7%)
	Urinary incontinence	64 (42.7%)
	Insomnia	83 (55.3%)
	Malnutrition	16 (10.7%)
	Delirium in the 6 months pre-admission	37 (24.7%)
	Delirium during hospitalization	68 (45.3%)
Morbidities, mean (SD)		5.76 (2.23)
Morbidities (number)	1–2	6 (4.0%)
	3–4	44 (29.3%)
	5 or more	100 (66.7%)
Charlson Index, mean (SD)		2.83 (2.19)
Frailty index (FI), mean (SD)		0.35 (0.15)
Frailty index, degrees	No frailty (0–0.19)	22 (14.6%)
	Mild frailty (0.20–0.35)	48 (32.0%)
	Moderate frailty (0.36–0.50)	55 (36.7%)
	Severe frailty (>0.50)	25 (16.7%)
Complexity identification	None	33 (22%)
	Complex chronic patients (CCPs)	85 (56.7%)
	Advanced chronic patients (ACPs)—end-of-life patients	32 (21.3%)
Therapeutic goal	Survival	29 (19.3%)
	Functionality	79 (52.7%)
	Symptomatic control	42 (28.0%)
**Baseline Pharmacological Data**		
Polypharmacy, mean (SD)		8.7 (3.8)
Polypharmacy, degrees	0–4 medications	25 (16.7%)
	5–9 medications	60 (40.0%)
	10 or more medications	65 (43.3%)
Medication Regimen Complexity Index (MRCI), mean (SD)		32.7 (16.1)
MRCI, degrees	Low complexity (0–19.99)	32 (21.4%)
	Moderate complexity (20–39.99)	71 (47.3%)
	High complexity (40 or more)	47 (31.3%)
Drug burden index (DBI), mean (SD)		1.04 (0.8)
DBI (degrees)	Low DBI (0–0.99)	76 (50.7%)
	Moderate DBI (1–1.99)	58 (38.7%)
	High DBI (2 or more)	16 (10.7%)
Inappropriate prescriptions (IP), mean (SD)		3.37 (2.56)
Number of IPs	0 IP	16 (10.7%)
	1 or more IPs	134 (89.3%)
	2 or more IPs	113 (75.3%)
	3 or more IPs	92 (61.3%)

* At this point, only patients living at home were assessed (*n* = 112), as nursing home patients’ medications were administered by staff.

**Table 2:** Relation between frailty and DBI scores.

Frailty Score	DBI Score	*p* Value *
Non-frail (0–0.19)	0.36 ± 0.48	
Mildly frail (0.2–0.35)	0.75 ± 0.59	<0.001
Moderately frail (0.36–0.50)	1.28 ± 0.73	
Severely frail (>0.50)	1.67 ± 0.81	

* *p*-value was calculated with ANOVA.

**Table 3:** Univariate and multivariate analyses pre-MR.

Frailty			
Patient Characteristics		Non-Frail (IF-VIG: 0.19) vs. Frail (IF-VIG: ≥0.20)	
		UnivariateOR (95% CI)	Multivariate OR (95% CI)
Age		0.93 (0.84–1.04)	1.04 (0.87–1.25)
Gender	Women	1.00	1
	Men	1.77 (0.65–4.81)	7.37 (1.50–36.15)
Origin	Home	1	na
	Nursing home	8.54 (1.11–65.81)	na
Barthel index		0.92 (0.88–0.95)	0.90 (0.85–0.95)
Barthel index	Independence: IB ≥ 95	1	na
	Mild dependence: IB 90–65	5.57 (1.83–16.95)	na
	Moderate dependence: IB 60–25	28.50 (5.72–142.01)	na
	Severe dependence: IB ≤ 20	-	na
Cognitive status	No dementia	1	na
	Mild dementia	8.48 (1.07–67.15)	na
	Moderate dementia (from GDS 5 to 6B)	-	na
	Advanced dementia (GDS 6C and above)	-	na
Geriatric syndromes		1.99 (1.37–2.90)	na
Geriatric syndromes	0	1	na
	1–2	4.07 (0.61–26,98)	na
	3 or more	26.40 (3.56–195.71)	na
Falls	No	1	na
	Yes	3.28 (1.05–10.26)	na
Depressive syndrome	No	1	na
	Yes	10.65 (2.39–47.44)	na
Insomnia	No	1	na
	Yes	4.03 (1.48–10.98)	na
Morbidities		1.50 (1.15–1.96)	na
Morbidities	1–2	1	na
	3–4	0.48 (0.05–4.49)	na
	5 or more	2.30 (0.24–22.16)	na
Chronicity	No chronicity	1	na
	Chronic complex patients (CCPs)	12.39 (4.23–36.30)	na
	End-of-life (EOL) patients	-	na
Therapeutic goal	Survival	1	na
	Functionality	8.28 (2.95–23.25)	na
	Symptomatic control	-	na
DBI		7.81 (2.89–21.13)	11.42 (2.77–47.15)

na: does not apply.

**Table 4:** Relation between cognitive status and degree of DBI.

Cognitive Status	DBI		
	0–0.99	1–1.99	≥2
No dementia	51 (69.9%)	18 (24.7%)	4 (5.5%)
Mild dementia	9 (40.9%)	13 (59.1%)	0 (0%)
Moderate dementia (from GDS5 to GDS 6B)	8 (30.8%)	15 (57.7%)	3 (11.5%)
Advanced dementia (GDS 6C and above)	8 (27.6%)	12 (41.4%)	9 (31.0%)

## Data Availability

The datasets generated and/or analysed during the current study are available from the corresponding author upon reasonable request.
